# Dose-Escalated Stereotactic Body Radiation Therapy for Prostate Cancer: Quality-of-Life Comparison of Two Prospective Trials

**DOI:** 10.3389/fonc.2016.00185

**Published:** 2016-08-29

**Authors:** Harvey C. Quon, Hima Bindu Musunuru, Patrick Cheung, Geordi Pang, Alexandre Mamedov, Laura D’Alimonte, Andrea Deabreu, Liying Zhang, Andrew Loblaw

**Affiliations:** ^1^University of Calgary, Calgary, AB, Canada; ^2^Tom Baker Cancer Centre, Calgary, AB, Canada; ^3^University of Toronto, Toronto, ON, Canada; ^4^Odette Cancer Centre, Sunnybrook Health Sciences Centre, Toronto, ON, Canada

**Keywords:** prostatic neoplasms, stereotactic body radiotherapy, radiotherapy, quality of life, clinical trial, radiation effects

## Abstract

**Introduction:**

The optimal prostate stereotactic body radiation therapy (SBRT) dose-fractionation scheme is controversial. This study compares long-term quality of life (QOL) from two prospective trials of prostate SBRT to investigate the effect of increasing dose (NCT01578902 and NCT01146340).

**Material and methods:**

Patients with localized prostate cancer received SBRT 35 or 40 Gy delivered in five fractions, once per week. QOL was measured using the Expanded Prostate Cancer Index Composite at baseline and every 6 months. Fisher’s exact test and generalized estimating equations were used to analyze proportions of patients with clinically significant change and longitudinal changes in QOL.

**Results:**

One hundred fourteen patients were included, 84 treated with 35 Gy and 30 treated with 40 Gy. Median QOL follow-up was 56 months [interquartile range (IQR) 46–60] and 38 months (IQR 32–42), respectively. The proportion of patients reporting clinically significant declines in average urinary, bowel, and sexual scores were not significantly different between dose levels, and were 20.5 vs. 24.1% (*p* = 0.60), 26.8 vs. 41.4% (*p* = 0.16), and 42.9 vs. 38.5% (*p* = 0.82), respectively. Similarly, longitudinal analysis did not identify significant differences in QOL between treatment groups.

**Conclusion:**

Dose-escalated prostate SBRT from 35 to 40 Gy in five fractions was not associated with significant decline in long-term QOL.

## Introduction

There has been significant interest in stereotactic body radiation therapy (SBRT) for prostate cancer, given the potential for increased tumor control (1), patient convenience, and lower treatment costs (2). However, the optimal dose and fractionation remain controversial, with total doses ranging from 33.5 to 50 Gy delivered in five fractions (3–5).

No randomized studies have been conducted to evaluate the impact of dose-escalated prostate SBRT. In the only prospective dose-escalation SBRT study to date, groups of 15 patients received 45, 47.5, and 50 Gy in five fractions (5). Grade 3+ toxicities were limited to one patient treated with 47.5 Gy and two patients who received 50 Gy. Bowel quality of life (QOL) was worse for patients on the 47.5 Gy arm, but no differences were found in urinary QOL.

Quality-of-life outcomes have become increasingly important in patient counseling as patient-reported experiences can be discordant from physician-rated toxicities (6). There is limited data examining QOL outcomes with increasing prostate SBRT dose. Therefore, we conducted a comparative analysis of long-term QOL outcomes from two prospective clinical trials to evaluate the impact of increasing prostate SBRT dose from 35 to 40 Gy in five fractions. Analysis of biochemical control and toxicity outcomes has been reported separately (7).

## Materials and Methods

This study is a secondary analysis of data from two prospective clinical trials (NCT01578902 and NCT01146340). Both trials were approved by the research ethics board at Sunnybrook Health Sciences Centre. Informed consent was provided by all patients.

### Study 1

Treatment details of study 1 have been previously published (3, 8). Men over 18 years of age with prostate adenocarcinoma and clinical stage T1-T2b (TNM 2002), Gleason sum ≤6, and Prostate-specific antigen (PSA) ≤ 10 ng/mL were eligible. Neoadjuvant androgen deprivation therapy (ADT) was permitted. Patients were excluded if they had received prior pelvic radiotherapy, a bleeding diathesis, which precluded gold fiducial marker insertion, hip prosthesis, pelvic girth > 40 cm, prostate > 90 cm^3^ on imaging, or International Prostate Symptom Score (IPSS) > 19.

Patients received 35 Gy in five fractions delivered once per week over 29 days. Each patient underwent transperineal insertion of three gold fiducial markers followed by CT simulation. Patients were immobilized in a vacuum lock bag (Vac-Lock, MED-TEC Inc., Orange City, IA, USA) with a comfortably full bladder and empty rectum for simulation and treatment.

The clinical target volume (CTV) consisted of the prostate only. Organs at risk (OAR) were contoured as solid organs and included the rectum, bladder, penile bulb, and femoral heads. The planning target volume (PTV) included the CTV plus an isotropic 4 mm margin. Planning objectives included the volume of CTV receiving 35 Gy (CTV V35 Gy) to receive >99%, PTV V33.25 Gy > 99%, PTV maximum dose (Dmax) ≤ 36.75 Gy. Normal tissue constraints were rectum V28 Gy ≤ 40%, V32 Gy ≤ 33%, bladder V32 Gy ≤ 40%, and penile bulb V20 ≤ 90%.

Patients were treated on linear accelerators (Siemens Primus, Concord, CA, USA; Elekta Synergy, Stockhold, Sweden) using a “step-and-shoot” intensity-modulated radiotherapy (IMRT) technique with 6 MV photons. Daily image-guidance was performed using orthogonal portal images to identify the fiducial markers and apply any necessary table shifts before treatment.

### Study 2

The second study included men over 18 years of age with prostate adenocarcinoma and clinical stage T1-2b, Gleason sum ≤ 6, and PSA ≤ 15 ng/mL; or clinical T1-2b, Gleason 7, and PSA ≤ 10 ng/mL. Neoadjuvant ADT was also permitted. Exclusion criteria were the same as for study 1.

Patients received 40 Gy in five fractions delivered once per week over 29 days. Patients underwent multiparametric MRI using cardiac surface coil with gadolinium infusion. After the MRI (to reduce artifacts), three gold fiducial markers were inserted transperineally. Patients, then, underwent CT simulation up to 1 week later. Immobilization and bladder and bowel filling were the same as for study 1. CT simulation images were fused with MRI for target delineation.

The CTV consisted of the prostate only and OAR were the same as for study 1. The PTV included the CTV and an isotropic 5 mm margin. Planning objectives included a CTV V40 Gy > 99%, PTV V38 Gy > 99%, and PTV Dmax ≤ 42 Gy. Normal tissue constraints were rectum V28 Gy ≤ 20% (up to maximum 40%), V31.8 Gy ≤ 15% (up to maximum 33%), bladder V31.8 Gy ≤ 15% (up to maximum 40%), and penile bulb V20 ≤ 90% (maximum V22.2 Gy < 90%). Patients were treated on the same machines using the same image-guidance protocol as for study 1.

### Patient Assessments

Quality of life was assessed using the Expanded Prostate Cancer Index Composite (EPIC) (9), a validated 50-item instrument that measures prostate cancer-specific QOL. It consists of four summary domains (urinary, bowel, sexual, and hormonal) with function and bother subscales for each domain. Scores were transformed to a 0–100 scale, with higher scores indicating better QOL.

Day 0 was defined as the start of radiotherapy. QOL was assessed at baseline and every 6 months thereafter. In study 2, QOL was also assessed at week 5 and month 3. Because QOL was not assessed during the acute time period in study 1, QOL comparisons will be restricted to late effects. Genitourinary (GU) and gastrointestinal (GI) toxicities were measured using the Common Terminology Criteria for Adverse Events Version 3 (CTCAE v3) during the acute period (≤3 months) and the Radiation Therapy Oncology Group (RTOG) late radiation morbidity schema for late effects (>3 months). Toxicities were assessed at baseline, weekly during treatment (only weeks 3 and 5 in study 2), at months 3 and 6, and every 6 months thereafter. PSA was assessed at baseline, months 3 and 6, and every 6 months. Follow-up for all QOL and toxicity endpoints continued for 5 years; biochemical and survival outcomes continued annually until the patient was discharged from follow-up.

### Statistical Analysis

Patient characteristics were summarized as median and interquartile range (IQR) for continuous variables, and proportions for categorical variables. The EPIC scores were calculated as mean ± SD and graphically presented as mean (with 95% confidence intervals) over time. A minimum clinically important change (MCIC) was defined as a decrease in QOL from baseline to follow-up, which exceeded half of the SD of that value at baseline (10). The “average EPIC change” was calculated by (mean of EPIC scores from month 6 to 60 – baseline score) while the “worst EPIC score” was calculated by (lowest of EPIC scores from month 6 to 60 – baseline score). These metrics were chosen to identify the average change in QOL as well as the maximum peak change in QOL, respectively. Actual, non-imputed data were used for analysis. Waterfall plots were created using changes in EPIC scores. In addition, to assess more severe changes in QOL, we compared the proportion of patients with 1 and 2 SD of change. Fisher’s exact test was used to compare categorical data between studies, including proportion of patients experiencing MCIC and proportion with moderate/big problems in specific EPIC items. Wilcoxon rank-sum test was used to compare continuous data. Two-sided *p*-value <0.05 was considered statistically significant. Generalized estimating equations methodology was used to account for repeated measurements and the impact of covariates on trends in EPIC scores over time (11), with binomial distribution and logit link function. All analyses were conducted using Statistical Analysis Software (SAS version 9.4 for Windows).

## Results

A total of 114 patients were included. There were 84 patients from study 1 and 30 from study 2 with median QOL follow-up of 56 months (IQR 46–60) and 38 months (IQR 32–42), respectively. Overall completion of EPIC questionnaires at 36 months was 70/95 (73.7%). Patient characteristics are described in Table 1 and were balanced between the groups with respect to age, clinical stage, baseline PSA, prostate volume, and baseline IPSS. Neoadjuvant ADT was given to one patient in study 1 and none in study 2. However, owing to different study eligibility criteria, there was a greater proportion of patients with Gleason 6 adenocarcinoma (100 vs. 60%, *p* < 0.0001) and Prostate Cancer Risk Stratification (ProCaRS) (12) low-risk disease (100 vs. 60%, *p* < 0.0001) in study 1.

**Table 1:** **Patient characteristics**.

	Study 1 (35 Gy/5 F) *N* = 84	Study 2 (40 Gy/5 F) *N* = 30	*p*-Value
Median age (IQR), years	67 (61–71)	68 (65–73)	0.27
Clinical stage			0.78
T1a-c	78 (92.9%)	27 (90.0%)	
T2a	6 (7.1%)	3 (10.0%)	
Median PSA at baseline (IQR), ng/mL	5.3 (4.2–7.3)	4.7 (3.5–7.5)	0.33
Gleason score			<0.0001
6	84 (100.0%)	18 (60.0%)	
7	0 (0%)	12 (40.0%)	
Risk group (ProCaRS) (12)			<0.0001
Low risk	84 (100%)	18 (60.0%)	
Low-intermediate risk	0 (0%)	11 (36.7%)	
High-intermediate risk	0 (0%)	1 (3.3%)	
Median TRUS prostate volume (IQR), mL	37 (29–55)	40 (31–53)	0.62
Median IPSS score (IQR)	5 (3–9)	5 (3–11)	0.66

*IQR, interquartile range; IPSS, International Prostate Symptom Score; TRUS, transrectal ultrasound*.

Baseline QOL data are shown in Data Sheet 1. There were no statistically significant differences in baseline EPIC summary domain scores between the two groups. However, there was a lower mean urinary function score in study 1 compared to study 2 (90.4 vs. 95.0%, *p* < 0.001).

The proportion of patients with average and worst changes in EPIC QOL score, which were clinically significant from months 6–60, are shown in Table 2. No statistically significant differences were found between treatment groups. MCIC was reported in average EPIC scores in 19.5 vs. 24.1% for urinary QOL (*p* = 0.60), 26.8 vs. 41.4% for bowel QOL (*p* = 0.16), and 42.9 vs. 38.5% in sexual QOL (*p* = 0.82) for the 35 and 40 Gy groups, respectively. When examining the worst change in EPIC QOL score, 67.1 vs. 58.6% had a urinary MCIC (*p* = 0.50), 65.9 vs. 62.1% had a MCIC in bowel score (*p* = 0.82), and 64.9 vs. 50.0% (*p* = 0.24) had MCIC in sexual QOL for study 1 and 2. Using additional cutoff values of 1 and 2 SD of baseline value to compare the proportion of patients with moderate or large changes in average QOL did not identify any significant differences (Data Sheet 2).

**Table 2:** **Proportion of patients with minimum clinically important change (MCIC)^a^ in EPIC quality-of-life scores by treatment group**.

	Average change			Worst change		
Summary domain	Study 1 (35 Gy/5 F)	Study 2 (40 Gy/5 F)	*p*-Value	Study 1 (35 Gy/5 F)	Study 2 (40 Gy/5 F)	*p*-Value
Urinary			0.60			0.50
No MCIC	66 (80.5%)	22 (75.9%)		27 (32.9%)	12 (41.4%)	
MCIC	16 (19.5%)	7 (24.1%)		55 (67.1%)	17 (58.6%)	
Bowel			0.16			0.82
No MCIC	60 (73.2%)	17 (58.6%)		28 (34.2%)	11 (37.9%)	
MCIC	22 (26.8%)	12 (41.4%)		54 (65.9%)	18 (62.1%)	
Sexual			0.82			0.24
No MCIC	44 (57.1%)	16 (61.5%)		27 (35.1%)	13 (50.0%)	
MCIC	33 (42.9%)	10 (38.5%)		50 (64.9%)	13 (50.0%)	

*QOL, quality of life; EPIC, Expanded prostate cancer index composite*.

*^a^Minimum clinically important change defined as a decrease in quality of life from baseline to follow-up which exceeds half of the standard deviation of the baseline value (10)*.

**Figure 1:**
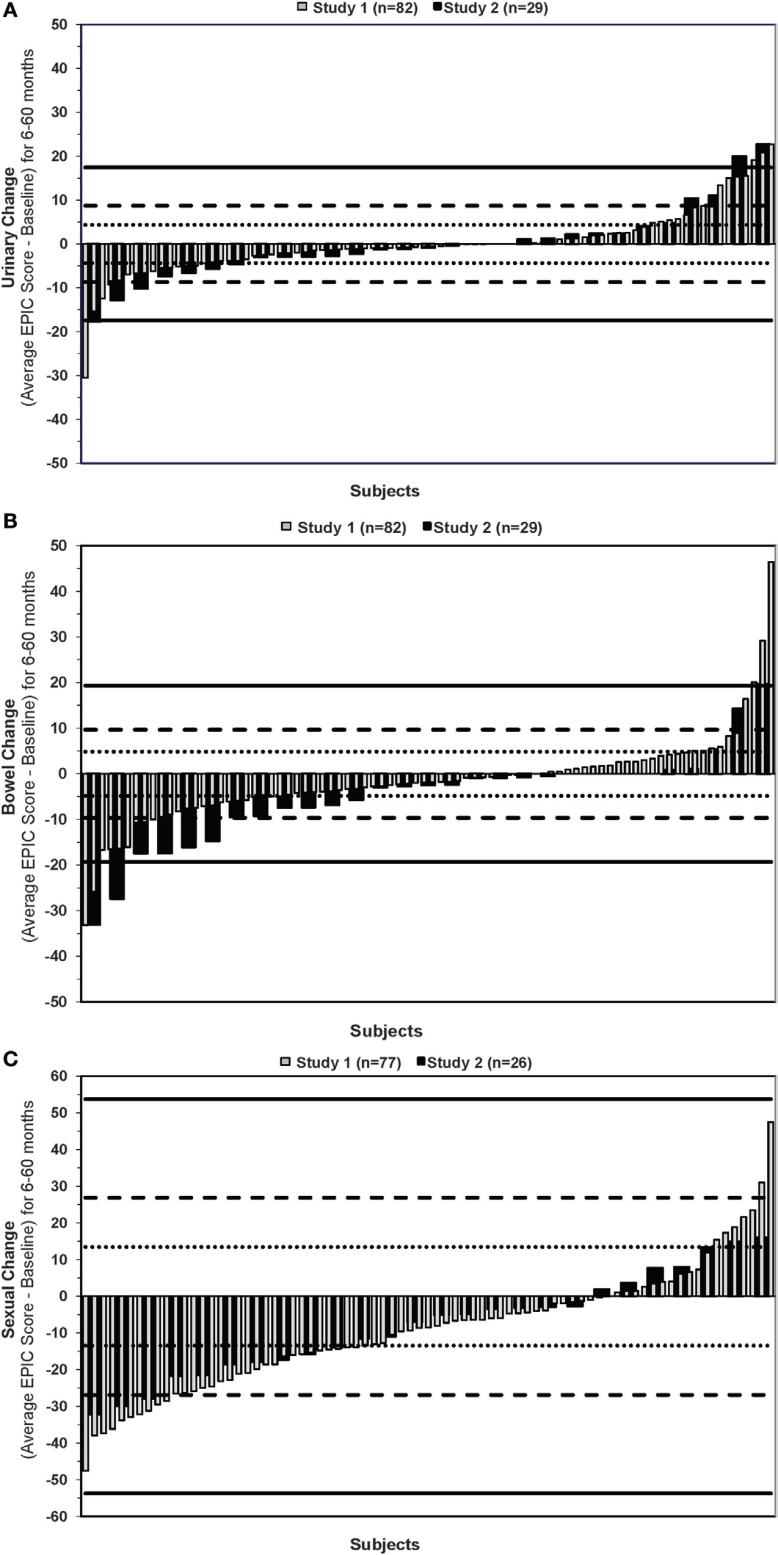
**Average change in EPIC (A) urinary (B) bowel and (C) sexual quality-of-life scores**. Negative and positive changes reflect worse and better QOL after treatment, respectively. Horizontal dotted, dashed, and straight lines indicate 0.5, 1, and 2 SD of the baseline score.

Waterfall plots of the average change in EPIC urinary, bowel, and sexual scores are shown in Figure 1.

### Analysis of QOL Over Time

**Figure 2:**
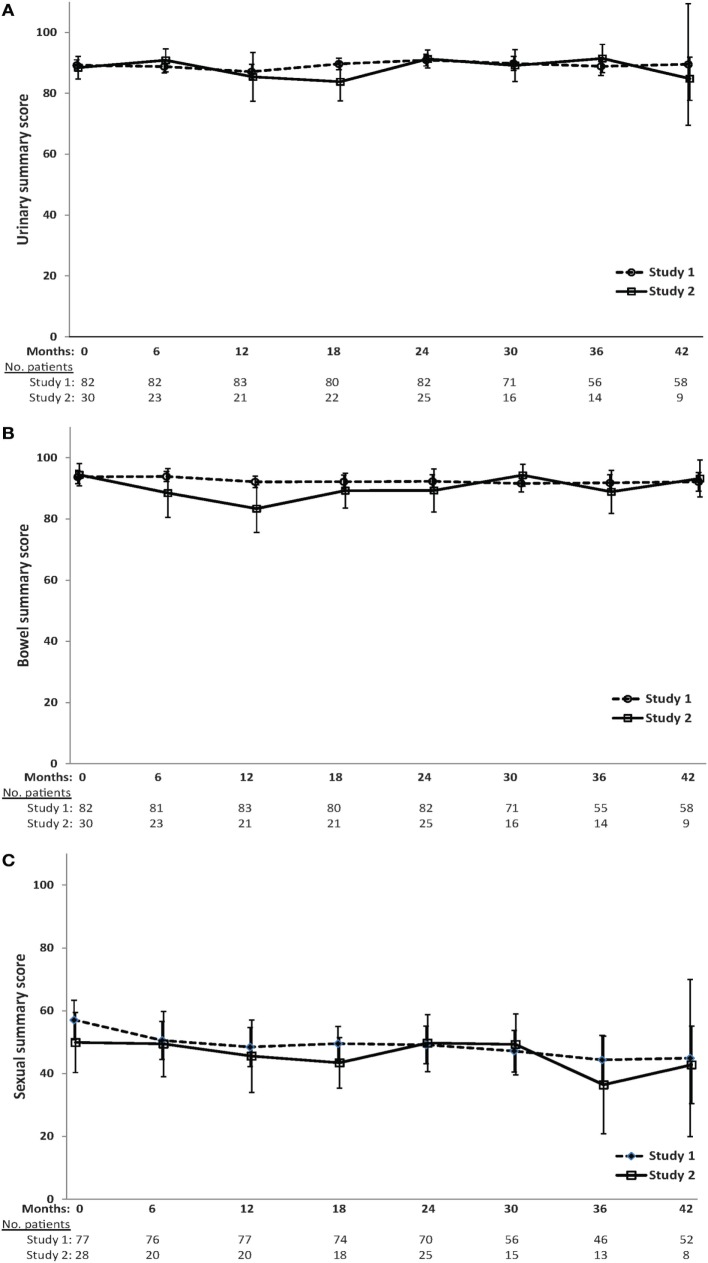
**Mean scores (and 95% confidence intervals) for (A) urinary (B) bowel (C) and sexual EPIC quality-of-life domains over time**.

Mean EPIC urinary, bowel, and sexual scores over time are shown in Figure 2. The QOL scores are fairly stable over time, and no significant differences exist between treatment groups at any time point, with the exception of lower bowel QOL for patients in study 2 at 6 (*p* = 0.06) and 12 months (*p* = 0.04) after treatment.

Longitudinal analysis of risk of MCIC in QOL by treatment group after adjustment for time is shown in Table 3. There was no significant time trend in MCIC of QOL scores with the exception of the sexual domain, in which there was an increasing risk of MCIC in sexual scores over time (OR 1.02, 95% CI 1.01–1.04, *p* < 0.001). This indicates a 2% increased risk of MCIC in sexual score per month. Treatment group was not significantly associated with MCIC in any of the QOL domains.

**Table 3:** **Longitudinal analysis of EPIC quality of life by treatment group**.

	OR	95% CI	*p*-Value
Urinary MCIC			
Time (months)	0.99	0.97–1.01	0.23
Treatment group (study 1 vs. 2)	0.98	0.54–1.79	0.95
Bowel MCIC			
Time (months)	1.00	0.99–1.02	0.99
Treatment group (study 1 vs. 2)	0.61	0.31–1.20	0.15
Sexual MCIC			
Time (months)	1.02	1.01–1.04	<0.001
Treatment group (study 1 vs. 2)	1.18	0.54–2.59	0.68

*OR, odds ratio; CI, confidence interval; MCIC, minimum clinically important change*.

The impact of baseline covariates on QOL scores was conducted while adjusting for time and treatment group. Analysis of urinary MCIC included the following covariates: baseline urinary QOL score (*p* = 0.01), age (years) (*p* = 0.18), clinical stage (T1a-c vs. T2a) (*p* = 0.63), log prostate volume (*p* = 0.73), Gleason score (6 vs. 7) (*p* = 0.21), log baseline PSA (*p* = 0.18), and log IPSS (*p* = 0.49). Only baseline urinary QOL score had a statistically significant effect (OR 1.05, 95% CI 1.01–1.10, *p* = 0.01), indicating that patients with higher baseline urinary score were at increased risk for MCIC. There was no significant interaction among the covariates.

Similarly, analysis of factors affecting bowel QOL included: baseline bowel QOL score (*p* = 0.18), age (years) (*p* = 0.84), clinical stage (T1a-c vs. T2a) (*p* = 0.13), log prostate volume (0.41), Gleason score (6 vs. 7) (*p* = 0.02), and log baseline PSA (*p* = 0.55). Only Gleason score was significant (OR 0.23, 95% CI 0.07–0.81, *p* = 0.02), with patients with Gleason 7 having lower risk of MCIC in bowel score compared to Gleason 6.

### Severity of QOL Impairment

Patients were asked “Overall, how big a problem has your (urinary function/bowel habits/sexual function) been for you during the last 4 weeks?” with answers dichotomized into no problem/very small problem/small problem vs. moderate problem/big problem. Results are listed in Table 4. There were no significant differences between the two studies at baseline or last follow-up. Few (<4%) patients reported moderate/severe problems in overall urinary function or bowel habits at last follow-up in both studies.

**Table 4:** **Overall problematic nature of quality-of-life domains at baseline and last follow-up**.

	Baseline *N* (%)			Last follow-up *N* (%)		
	Study 1	Study 2	p*	Study 1	Study 2	p*
Urinary function			0.39			0.98
No/very small/small problem	77 (95.1)	27 (90.0)		81 (96.4)	28 (96.6)	
Moderate/severe problem	4 (4.9)	3 (10.0)		3 (3.6)	1 (3.5)	
Bowel habits			0.45			0.98
No/very small/small problem	80 (98.8)	28 (96.6)		81 (96.4)	28 (96.6)	
Moderate/severe problem	1 (1.2)	1 (3.5)		3 (3.6)	1 (3.5)	
Sexual function			0.91			0.92
No/very small/small problem	60 (76.9)	22 (75.9)		60 (74.1)	21 (75.0)	
Moderate/severe problem	18 (23.1)	7 (24.1)		21 (25.9)	7 (25.0)	

*Comparison between study 1 and study 2.

## Discussion

This is one of the first studies to examine the effect of dose-escalation in prostate SBRT on QOL endpoints. The results show that when increasing the dose from 35 to 40 Gy, delivered in five fractions once per week, there is no significant difference in long-term QOL outcomes. Unique features of this analysis include prospective data collection and longer follow-up.

The results of the present study should be compared to existing literature within the context of differences in treatment technique and total dose. There is conflicting data regarding the impact of conventionally fractionated, dose-escalated radiotherapy on QOL endpoints using older radiation techniques (13–16). In the Proton Radiation Oncology Group 9509 randomized study of 70.2 vs. 79.2 Gy, there were no differences in urinary, bowel, or sexual outcomes using the Prostate Cancer Symptom Indices assessed at a median 9.4 years (13). Similarly, in the GETUG-06 trial of 70 vs. 80 Gy, there were no differences in QOL using the European Organization for Research and Treatment of Cancer (EORTC) QOL Questionnaire and prostate-specific module (14).

In contrast, other studies have shown detriment in specific patient-reported outcomes after dose-escalated radiotherapy. In the MD Anderson randomized trial of 70 vs. 78 Gy, there was a significant increase in frequency of bowel movements associated with high-dose radiotherapy at 3 years after treatment (*p* = 0.03) (15). No differences were found in the other bowel-related, urinary, or sexual QOL. Also, in a detailed analysis of GI outcomes from the UK RT01 randomized trial comparing 64 vs. 74 Gy, QOL assessment using the University of Los Angeles Prostate Cancer Index showed no differences in diarrhea, bowel distress, or bowel problems (16). However, there was an increased risk of moderate abdominal pain (HR 1.53, 95% CI 1.13–2.06) and severe rectal urgency (HR 1.64, 95% CI 1.11–2.42) observed with higher dose.

Many studies have reported on QOL outcomes after prostate SBRT (5, 17), but few have compared such data with respect to the effect of increasing dose. The previously mentioned dose-escalation study of 45, 47.5, and 50 Gy in five fractions was not powered to compare QOL outcomes between treatment arms (5). However, comparison of QOL measured by the EPIC instrument did not identify any difference in bowel QOL between the 45 and 50 Gy arms (*p* = 0.5), but there was significantly worse bowel QOL for the 47.5 Gy dose level (*p* = 0.01). No differences were found in urinary QOL between the three dose levels.

In a separate analysis of physician-rated toxicities from the present study, dose-escalated SBRT was associated with greater risk of grade 2+, but not grade 3+, toxicities (7). Other studies have found similar associations between higher SBRT dose and toxicities (18), and there are several possible explanations for why it did not result in worse patient-reported outcomes (19), in the present study. First, the increase in biologically effective dose to OAR is dependent upon the alpha–beta ratio. For the rectum, the alpha–beta ratio for grade≥2 late toxicity has been estimated to be 4.8 Gy, but with a wide 68% CI ranging from 0.6 to 46 Gy (20). The corresponding difference between dose levels in the present study in equivalent 2 Gy fractions would be 14.6 Gy (68% CI 6.4–30.0), but may be as low as 6.4 Gy, if the alpha–beta ratio for bowel QOL is similar to that for late toxicity. Second, the relationship between dose and QOL outcomes has not been well characterized, and differences in dose at 35 to 40 Gy may not be expected to result in significant changes in QOL (i.e., on the “flat” segment of the dose-complication curve). Third, although this study used the validated EPIC instrument and commonly reported threshold of one-half SD (10) to identify a MCIC, it may not have been sensitive enough to detect small changes in QOL. Finally, patients who received higher dose may have had worse treatment-related toxicity, but adapted to the effects of altered function, and not reported any impairment on the QOL questionnaires (21).

The QOL outcomes reported in this study are similar to those reported by other groups. In a multi-institutional study of 864 patients treated with prostate SBRT to a median dose of 36.25 Gy in four or five fractions (17), EPIC scores were similar to the present study with long-term urinary, bowel, and sexual scores of 80–90, 85–95, and 20–50. Although 20–40% of patients in our study reported a change in long-term QOL, which was clinically detectable, the majority of changes were mild to moderate in severity. This is a limitation of the MCIC definition in that it does not distinguish minimal from severe changes in QOL.

To assess severe problems in QOL, we also analyzed specific EPIC items, which asked about overall patient function. The proportion of patients who reported moderate or severe problems in overall urinary function or bowel habits was <4% at both dose levels, which is reassuring. Also while 25% of patients experienced moderate or severe problems in sexual function at last follow-up, this is similar to values observed at baseline.

We analyzed the effect of patient, tumor, and treatment-related factors to identify variables associated with worse post-treatment QOL. Only a higher baseline urinary QOL score was associated with greater risk of clinically significant change in QOL after treatment. This finding is similar to other QOL studies in which patients with greater current function have more potential for loss of function, compared to patients with intermediate or poor baseline function (22). Gleason 7 was also found to be associated with greater risk of MCIC in bowel QOL. The reason for this is unclear and requires further investigation, but may be a type I error.

Limitations of our study should be noted. As QOL was not assessed in study 1 during the first 3 months after treatment, acute changes in QOL could not be compared. Also, the smaller sample size of patients receiving 40 Gy limited the power to detect small differences between study groups. We have completed accrual to a multicentre, phase 2 randomized trial of 40 Gy in five fractions, delivered every other day vs. once weekly (23). One hundred fifty-two patients had acute and late QOL measured, and we will report these data as they mature. Finally, radiotherapy planning was different between the two cohorts. CTV volumes were based on CT and MRI for patients receiving 40 Gy, whereas it was based on CT only imaging for those treated with 35 Gy. Offset against this, the PTV margin was 5 mm for the 40 Gy study and 4 mm for the 35 Gy study. Compared to CT-based planning, MRI-defined prostate volumes are 21–30% (24–26) smaller, which has been shown to reduce rectal and bladder doses (26). However, these dosimetric advantages have only translated into reduced urinary, but not rectal, toxicity in reports with clinical outcomes (24, 26). No studies have compared QOL endpoints with MRI vs. CT-based planning.

## Conclusion

Increased dose for prostate SBRT from 35 to 40 Gy, delivered in five once-weekly fractions, was not associated with a clinically significant difference in late QOL outcomes. Long-term effects on urinary and bowel QOL remain mild to moderate, with most patients reporting the same QOL scores in late follow-up compared to baseline.

## Author Contributions

HQ, HM, and AL participated in the study conception and design, data analysis, and interpretation of results. PC, GP, and LZ were involved in the data analysis and interpretation. AM, LD, and AD participated in the collection and assembly of data. All authors were involved in writing and final approval of the manuscript.

## Conflict of Interest Statement

The authors declare that the research was conducted in the absence of any commercial or financial relationships that could be construed as a potential conflict of interest.

## Abbreviations

ADT, androgen deprivation therapy; CTV, clinical target volume; Dmax, maximum dose; EORTC, European Organization for Research and Treatment of Cancer; EPIC, Expanded Prostate Cancer Index Composite; GI, gastrointestinal; GU, genitourinary; HR, hazard ratio; IMRT, intensity-modulated radiotherapy; IPSS, International Prostate Symptom Score; IQR, interquartile range; MCIC, minimum clinically important change; OAR, organs at risk; ProCaRS, prostate cancer risk stratification; PSA = prostate-specific antigen; PTV, planning target volume; QOL, quality of life; RTOG, Radiation Therapy Oncology Group; SBRT, stereotactic body radiation therapy.
