# Supplementary material for: Dose-Escalated Stereotactic Body Radiation Therapy for Prostate Cancer: Quality-of-Life Comparison of Two Prospective Trials
Source: Front Oncol. 2016 Aug 29;6:185. doi: 10.3389/fonc.2016.00185 (PMC5002986; doi:10.3389/fonc.2016.00185)
Supplement: Supplementary file 1 [file Data_Sheet_1.DOC]

Appendix A. Baseline EPIC quality of life scores by treatment group

| **Summary domain** | **Study 1**  **(35 Gy/5 F)** | **Study 2**  **(40 Gy/5 F)** | **p-value** |
| --- | --- | --- | --- |
| Urinary | 89.2 (8.3) | 88.4 (10.0) | 0.72 |
| Urinary Function | 90.4 (6.2) | 95.0 (7.3) | <0.001 |
| Urinary Bother | 88.4 (12.1) | 83.8 (13.5) | 0.09 |
| Bowel | 93.7 (9.7) | 94.5 (9.7) | 0.23 |
| Bowel Function | 93.5 (8.2) | 94.9 (8.4) | 0.13 |
| Bowel Bother | 94.0 (12.9) | 94.1 (12.0) | 0.55 |
| Sexual | 57.1 (27.5) | 49.9 (24.6) | 0.13 |
| Sexual Function | 50.4 (27.3) | 45.5 (25.2) | 0.31 |
| Sexual Bother | 72.4 (34.3) | 63.2 (33.0) | 0.17 |

Values are mean (standard deviation).

Appendix B. Proportion of patients with small, moderate, and large changes in average EPIC quality of life scores by treatment group

|  | Study 1  (35 Gy/5 F)  N=82 | Study 2  (40 Gy/5F)  N=29 | p-value |
| --- | --- | --- | --- |
| Small improvement or decline |  |  |  |
| Urinary |  |  |  |
| > +0.5 SD | 15 (18.29%) | 6 (20.69%) | 0.79 |
| < -0.5 SD | 16 (19.51%) | 7 (24.14%) | 0.60 |
| Bowel |  |  |  |
| > +0.5 SD | 10 (12.20%) | 3 (10.34%) | 1.0 |
| < -0.5 SD | 22 (26.83%) | 12 (41.38%) | 0.16 |
| Moderate improvement or decline |  |  |  |
| Urinary |  |  |  |
| > +1 SD | 8 (9.76%) | 4 (13.79%) | 0.51 |
| < -1 SD | 4 (4.88%) | 3 (10.34%) | 0.37 |
| Bowel |  |  |  |
| > +1 SD | 4 (4.88%) | 2 (6.90%) | 0.65 |
| < -1 SD | 8 (9.76%) | 6 (20.69%) | 0.19 |
| Large improvement or decline |  |  |  |
| Urinary |  |  |  |
| > +2 SD | 3 (3.66%) | 2 (6.90%) | 0.60 |
| < -2 SD | 1 (1.22%) | 1 (3.45%) | 0.46 |
| Bowel |  |  |  |
| > +2 SD | 3 (3.66%) | 1 (3.45%) | 1.0 |
| < -2 SD | 2 (2.44%) | 2 (6.90%) | 0.28 |
